# A Novel Mono-surface Antisymmetric 8Tx/16Rx Coil Array for Parallel Transmit Cardiac MRI in Pigs at 7T

**DOI:** 10.1038/s41598-020-59949-6

**Published:** 2020-02-20

**Authors:** Ibrahim A. Elabyad, Maxim Terekhov, David Lohr, Maria R. Stefanescu, Steffen Baltes, Laura M. Schreiber

**Affiliations:** Chair of Cellular and Molecular Imaging, Comprehensive Heart Failure Center (CHFC), University Hospital Wuerzburg, D-97078 Wuerzburg, Germany

**Keywords:** Biomedical engineering, Engineering

## Abstract

A novel mono-surface antisymmetric 16-element transmit/receive (Tx/Rx) coil array was designed, simulated, constructed, and tested for cardiac magnetic resonance imaging (cMRI) in pigs at 7 T. The cardiac array comprised of a mono-surface 16-loops with two central elements arranged anti-symmetrically and flanked by seven elements on either side. The array was configured for parallel transmit (pTx) mode to have an eight channel transmit and 16-channel receive (8Tx/16Rx) coil array. Electromagnetic (EM) simulations, bench-top measurements, phantom, and MRI experiments with two pig cadavers (68 and 46 kg) were performed. Finally, the coil was used in pilot *in-vivo* measurements with a 60 kg pig. Flip angle (FA), geometry factor (g-factor), signal-to-noise ratio (SNR) maps, and high-resolution cardiac images were acquired with an in-plane resolution of 0.6 mm × 0.6 mm (*in-vivo*) and 0.3 mm × 0.3 mm (*ex-vivo*). The mean g-factor over the heart was 1.26 (R = 6). Static phase ${B}_{1}^{+}$ shimming in a pig body phantom with the optimal phase vectors makes possible to improve the ${B}_{1}^{+}$ homogeneity by factor > 2 and transmit efficiency by factor > 3 compared to zero phases (before RF shimming). Parallel imaging performed in the *in-vivo* measurements demonstrated well preserved diagnostic quality of the resulting images at acceleration factors up to R = 6. The described hardware design can be adapted for arrays optimized for animals and humans with a larger number of elements (32–64) while maintaining good decoupling for various MRI applications at UHF (e.g., cardiac, head, and spine).

## Introduction

With the development of ultrahigh field (UHF) strength (${B}_{0}$ ≥ 7 T) magnetic resonance imaging (MRI) scanners, a significant improvement in the SNR, and hence in spatial and temporal resolutions can be achieved compared to conventional lower field strength (e.g., ${B}_{0}\,$≤ 3 T) MRI scanners^1,2^. Because of the radiofrequency (RF) wavelength-lowering effects of the ${B}_{1}^{+}$ field at 7 T (${{\rm{\lambda }}}_{{\rm{eff}}}\,$≈ 12 cm, i.e., in the order of a human thorax), the design of an optimized RF coil array at 7 T is becoming more challenging. This is because of standing waves creating constructive and destructive interferences of the transmitted ${B}_{1}^{+}$ field magnitude and hence strong intensity artifacts occurring in the acquired MR images^3–6^. For brain imaging at 7 T, different coil array designs and technologies have been introduced to solve the issues of ${B}_{1}^{+}$ field inhomogeneity such as microstrip transmission line (MTL) resonators^7–12^, inverted (MTL) resonators^13^, stepped impedance resonators^14–19^, and by utilizing a high-impedance surface as the RF-shield to improve the efficiency and penetration of the ${B}_{1}^{+}$ field^20,21^.

Despite the numerous technical challenges related to ${B}_{0}\,\,$and ${B}_{1}^{+}$ field inhomogeneities, the application of UHF scanners for cardiovascular research holds significant promise^22,23^. Another hardware challenge is the necessity to increase the number of elements of the array at 7 T (typical 16–32) to allow for parallel imaging and RF-shimming while keeping all resonant coil elements well decoupled. At 7 T, most of the commercial human cardiac coil arrays are designed as local Tx/Rx arrays. Different coil arrays and design concepts have been introduced for cMRI in humans at UHF including, MTL resonators^24,25^, conventional multichannel local Tx/Rx loop arrays^26–32^, dipole antenna arrays^33–39^, combined dipoles and loop arrays^40,41^ and dielectric resonant antenna arrays^42^.

The existing commercial cardiac coil arrays for humans at 7 T comprise two independent anterior and posterior arrays. The anterior array is the most efficient, because it is fixed near to the heart region and its element dimensions are optimized to have good penetration and transmit efficiency (${{\rm{Tx}}}_{{\rm{eff}}}$) of the ${B}_{1}^{+}$ field up to 10 cm depth within the thorax. The posterior array is used in order to improve the penetration of the ${B}_{1}^{+}$ field within the heart from the back side of the thorax. However, the contribution of the ${B}_{1}^{+}$ field from the posterior array is low compared to the anterior array due to its large distance to the heart. Recent studies^43,44^ presented two 8Tx/16Rx coil arrays comprising two independent anterior−posterior (A–P) parts optimized for moderate weight pigs (<50 kg). The distance from the center of the pig heart to the posterior array for the 46 kg pig was about 16 cm and, thus, the posterior array contributed only about 10% of the total ${B}_{1}^{+}$ field, despite the optimized sizes of the array elements.

The magnitude and phase of the signal for each individual transmit coil element can be optimized to provide a uniform combined ${B}_{1}^{+}$ field distribution in the selected region-of-interest (ROI). This process is referred to as RF-shimming or ${B}_{1}^{+}$ shimming^45–50^. Using eight independent RF power amplifiers (RFPAs) in pTX mode allows driving the individual 8Tx channels of the array dynamically (i.e. to vary the magnitude/phase of each channel or the RF-pulse waveforms), which enables shaping of 2D and 3D excitation profiles based on a wide-range of optimization criteria^51,52^. Another major issue is that the limited RF power given from the RFPA is distributed to both array parts. Approximately half of the RF power contributes to only 10% of the total ${B}_{1}^{+}$ field under the assumption that the power is divided equally to both arrays. This makes the standard vendor-integrated pTx ${B}_{1}^{+}$ shimming algorithm, which is usually developed for the cylindrical Tx/Rx head coils (e.g., birdcage) inefficient for cardiac arrays, comprising two independent anterior and posterior arrays. The vendor-integrated pTx ${B}_{1}^{+}$ shimming algorithm targeted to achieve an optimal combination of ${B}_{1}^{+}$ field homogeneity and mean value, tries to compensate the low penetration from the posterior array, and thus provides high amplitudes to most of its channels, resulting in low amplitudes for some of the channels of the anterior array.

One of the major challenges associated in cMRI is gating the acquisition to compensate the heart motion and that of surrounding tissues such as the lung. Standard cMRI protocols actively implement the parallel acquisition techniques (PAT), which allows for reducing total acquisition time (TA), leading, however, to an SNR penalty proportional to the square root of the phase encoding lines reduction factor and g-factor of the array. Therefore, characterization of the noise amplification (g-factor) is an important aspect when testing RF coil arrays for cMRI at 7 T. The rationale of proposed mono-surface antisymmetric array design for 7 T cMRI in pigs is to reduce the coupling among the elements and to provide optimal receive properties for efficient parallel acquisition, while simultaneously minimizing the mutual correlation of the transmitted ${B}_{1}^{+}$ fields generated by individual elements and, hence, to improve RF-shimming capabilities. This mono-surface array design combines the properties of both volume resonator and surface coils in the most appropriate way and provides a well-balanced of combination of both ${B}_{1}^{+}$ and ${B}_{1}^{-}$ fields.

In this paper, we report a novel 8Tx/16Rx pTx cardiac coil array composed of 16-loop elements allocated on the mono-surface of one printed circuit board (PCB) and fixed on half-elliptical housing. The housing dimensions were designed to fit with pigs of body weights ranging from 40 to 80 kg. Final tuning and matching of the mono-surface array were done using a 46 kg pig cadaver. The testing and validation of the array’s transmitting and receiving properties was done using two cadaver pigs (68 and 46 kg) in weights. Finally, the cardiac array was used in the *in-vivo* scans using a 60 kg pig. The mean g-factor within the heart region of 1.26 was shown with an acceleration factor of R = 6. Static phase ${B}_{1}^{+}$ shimming in a pig body phantom with the optimal phase vectors shows the potential to improve the ${B}_{1}^{+}$ homogeneity characterized via relative-standard-deviation (RSD) by factor > 2 and transmit efficiency (${{\rm{Tx}}}_{{\rm{eff}}}$) by factor > 3 compared to zero-phases (without RF-shimming). To investigate the potential benefit of the mono-surface array in pTx ${B}_{1}^{+}$ shimming and parallel imaging, phantom and cadaver pig MR-measurements were additionally performed and compared to an 8Tx/16Rx pTx human cardiac array prototype. After ${B}_{1}^{+}$ shimming using the vendor pTx algorithm, the mono-surface array has demonstrated improvement in ${B}_{1}^{+}$ homogeneity coefficient by factor > 4 in a 20 cm spherical phantom and by about 43% in a pig body phantom compared to before shimming with hardware (HW) phases.

## Results

### Array characterization

#### S-matrix measurements

Simulated and measured S-matrices for the mono-surface array loaded with the pig body phantom are shown in Table 1. Simulated and measured S-matrices for the mono-surface array loaded with a 20 cm diameter spherical phantom are shown in Supplementary Table S1. For the spherical phantom, the worst-case transmission coefficient ${{\rm{S}}}_{{\rm{ij}}}$ was below −8 dB due to reduced loading of the side elements (e.g., 14 and 16). For the pig body phantom, the worst-case transmission coefficient ${{\rm{S}}}_{{\rm{ij}}}$ was below −10 dB between elements 14 and 16. The S-matrix was measured when the array was loaded with a cadaver of 46 kg pig (cadaver #2) [see Supplementary Table S2]. With loading of the *ex-vivo* pig, reflection coefficients ${{\rm{S}}}_{{\rm{ii}}}$ were below −15 dB for all 16 elements. The worst-case transmission coefficient ${{\rm{S}}}_{{\rm{ij}}}$ was below −11 dB between elements 1 and 6 and between elements 2 and 5.

**Table 1:** Simulated (italic) and measured S-Matrix in dB for the mono-surface array loaded with the pig body phantom with ${{\rm{\varepsilon }}}_{{\rm{r}}}$ = 59.3 and $\sigma$ = 0.79 S/m.

El.#	1	2	3	4	5	6	7	8	9	10	11	12	13	14	15	16	El.#
	**−13**	−16	−20	−15	−20	−15	−28	−26	−23	−20	−35	−29	−23	−25	−27	−36	1
		**−14**	−14	−22	−14	−23	−20	−28	−17	−23	−33	−36	−21	−23	−30	−24	2
1	**−16**		**−21**	−22	−16	−30	−12	−29	−24	−28	−19	−44	−23	−27	−30	−30	3
2	*−17*	**−16**		**−21**	−30	−16	−38	−11	−28	−12	−48	−17	−30	−27	−38	−28	4
3	*−21*	*−12*	**−18**		**−24**	−20	−15	−27	−11	−27	−20	−27	−19	−27	−28	−30	5
4	*−12*	*−21*	*−27*	**−18**		**−20**	−24	−18	−24	−13	−29	−24	−27	−20	−34	−19	6
5	*−18*	*−14*	*−15*	*−28*	**−15**		**−15**	−32	−16	−45	−12	−34	−17	−37	−17	−38	7
6	*−14*	*−19*	*−28*	*−15*	*−22*	**−15**		**−14**	−34	−12	−34	−11	−34	−13	−48	−14	8
7	*−25*	*−35*	*−12*	*−36*	*−15*	*−27*	**−21**		**−24**	−27	−20	−35	−11	−30	−12	−42	9
8	*−35*	*−25*	*−36*	*−12*	*−27*	*−15*	*−26*	**−21**		**−24**	−35	−19	−38	−13	−50	−12	10
9	*−18*	*−17*	*−13*	*−25*	*−13*	*−22*	*−19*	*−24*	**−28**		**−15**	−42	−10	−35	−22	−42	11
10	*−17*	*−18*	*−25*	*−13*	*−22*	*−13*	*−24*	*−18*	*−20*	**−28**		**−16**	−37	−10	−37	−14	12
11	*−24*	*−26*	*−22*	*−36*	*−25*	*−29*	*−10*	*−33*	*−18*	*−32*	**−21**		**−16**	−33	−11	−37	13
12	*−26*	*−24*	*−36*	*−22*	*−29*	*−25*	*−33*	*−10*	*−32*	*−19*	*−42*	**−20**		**−27**	−44	−10	14
13	*−22*	*−21*	*−23*	*−32*	*−31*	*−29*	*−12*	*−27*	*−10*	*−27*	*−10*	*−37*	**−15**		**−13**	−41	15
14	*−21*	*−22*	*−32*	*−23*	*−30*	*−31*	*−27*	*−12*	*−26*	*−10*	*−37*	*−10*	*−32*	**−15**		**−13**	16
15	*−36*	*−56*	*−22*	*−36*	*−24*	*−47*	*−16*	*−42*	*−10*	*−35*	*−23*	*−41*	*−10*	*−39*	**−15**		
16	*−57*	*−36*	*−36*	*−22*	*−47*	*−24*	*−41*	*−16*	*−35*	*−10*	*−41*	*−23*	*−39*	*−10*	*−45*	**−15**	
	1	2	3	4	5	6	7	8	9	10	11	12	13	14	15	16	

#### Quality factor measurements

Since the mono-surface array has different element dimensions, bending, and loading conditions due to the heterogeneity of the pig thorax, the quality factors (${\rm{Q}}$) will be different. Both unloaded (${{\rm{Q}}}_{{\rm{un}}}$) and loaded (${{\rm{Q}}}_{{\rm{lo}}}$) quality factors were measured from matched ${{\rm{S}}}_{{\rm{ii}}}$ reflection coefficients for all 16 loops. For all 16-elements, ${{\rm{Q}}}_{{\rm{lo}}}$ were measured when the array loaded with a 46 kg pig cadaver. The measured ${{\rm{Q}}}_{{\rm{lo}}}/{{\rm{Q}}}_{{\rm{un}}}$ ratio for all elements was in the range of 0.50 to 0.75.

### Phantom MRI measurements

#### Spherical phantom

**Figure 1:**
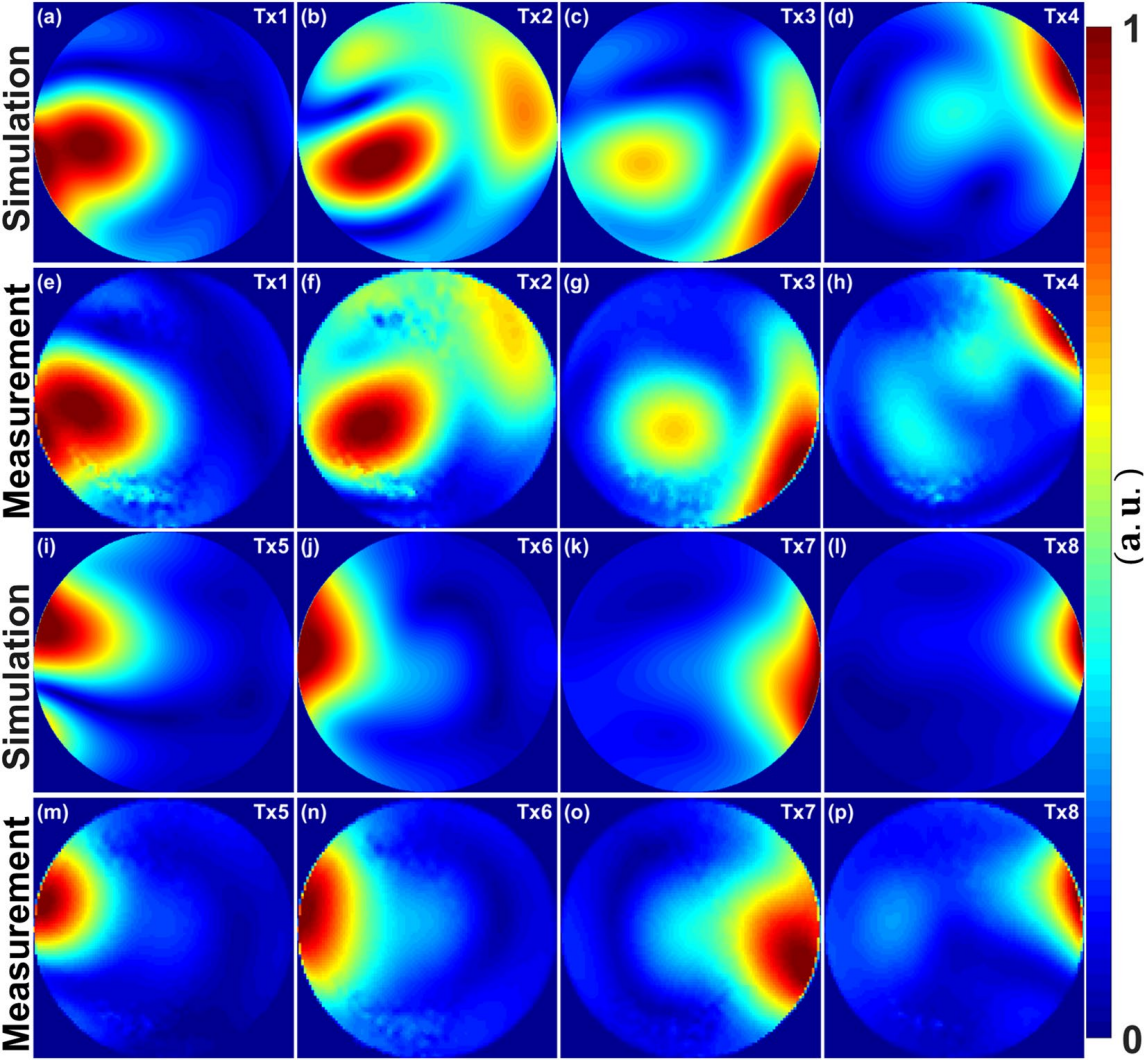
Simulated normalized central coronal ${B}_{1}^{+}$ field distributions and measured FA maps for the individual 8Tx channels (${T}_{x1}$**–**${T}_{x8}$) of the mono-surface array within a spherical phantom of 20 cm in diameter. Simulation (**a**–**d**) & (**i**–**l**). Measurement (**e**–**h**) & (**m**–**p**). All images were seen from F–H direction as shown in the array setup of Fig. 4 (**h**).

Figure 1 illustrates the simulated and measured central coronal ${B}_{1}^{+}$ field distributions within a 20 cm diameter spherical phantom for eight individual Tx-channels (${T}_{x1}$, ${T}_{x2}$, ${T}_{x3}$, ${T}_{x4}$, ${T}_{x5}$, ${T}_{x6}$, ${T}_{x7}$, and ${T}_{x8}$). The combined ${B}_{1}^{+}$ fields per channel were normalized to their maxima. Good agreement between the CST simulations and the experimentally measured FA maps was achieved for most of the channels (${T}_{x1}$, ${T}_{x2}$, ${T}_{x3}$, ${T}_{x6}$, ${T}_{x7}$, and ${T}_{x8}$). The ${B}_{1}^{+}$ field patterns show good similarity between simulations and measurements and match well even within the locations of destructive interferences. However, some differences between simulated and measured FA maps are observed for the Tx-channels (${T}_{x4}$ and ${T}_{x5}$) [see Fig. 1(d,h,i,m)].

### Pig body phantom

**Figure 2:**
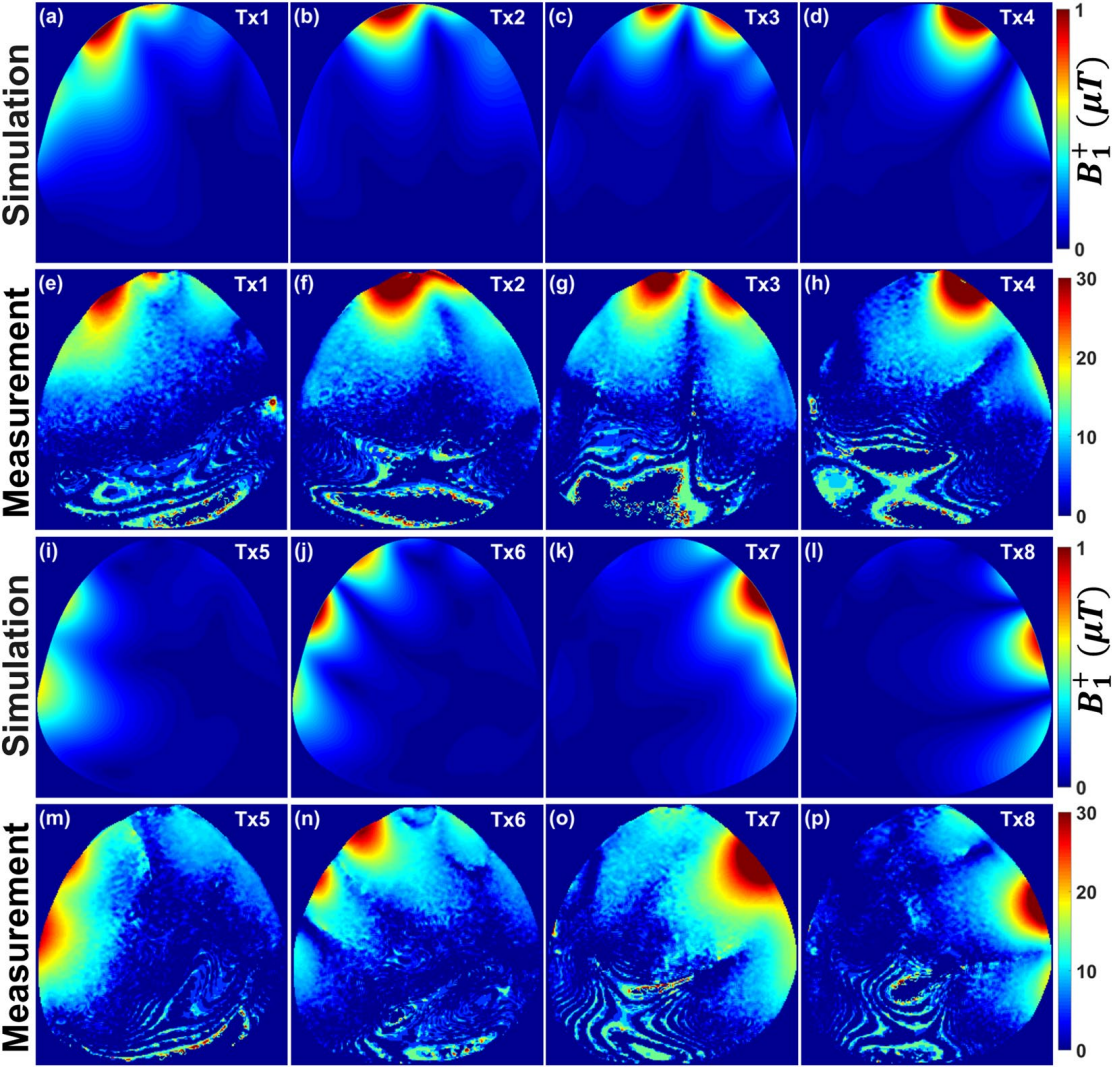
Simulated central transversal ${B}_{1}^{+}$ field distribution in $\mu {\rm{T}}$ and measured FA maps in degrees for the individual 8Tx channels (${T}_{x1}$**–**${T}_{x8}$) of the mono-surface array within the pig body phantom. Simulation (**a**–**d**) & (**i**–**l**). Measurement (**e**–**h**) & (**m**–**p**). All images were seen from F–H direction as shown in the array setup of Fig. 4 (**h**).

Figure 2 demonstrates the simulated ${B}_{1}^{+}$ field distribution in the central transversal cross-section and the measured in corresponding position FA maps within the pig body phantom. Good agreement between the simulated ${B}_{1}^{+}$ field distributions and the experimentally measured FA maps was achieved for all 8Tx channels.

### Validation of ${{\boldsymbol{B}}}_{1}^{+}$ shimming (combined FA maps)

**Figure 3:**
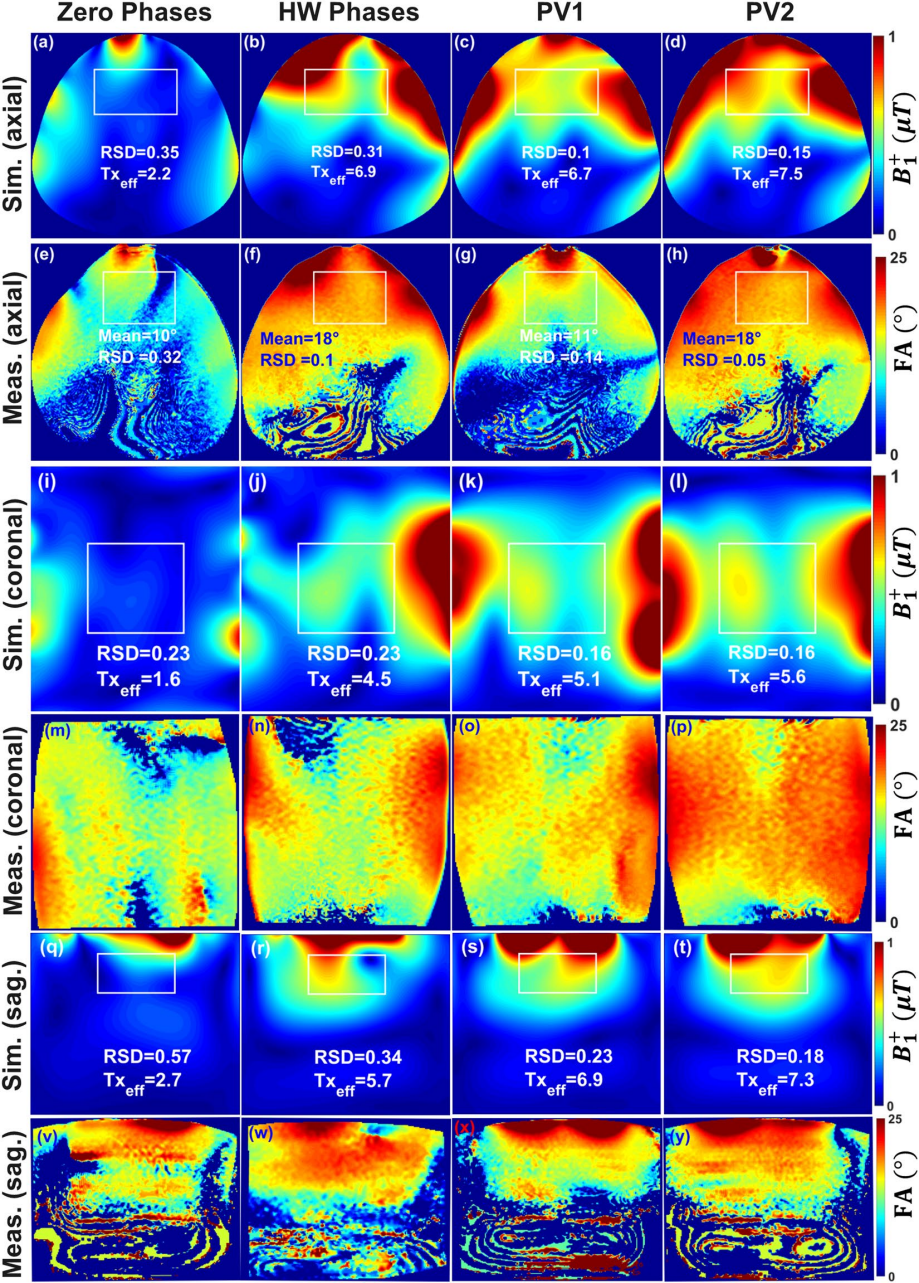
Simulated combined ${B}_{1}^{+}$ field distribution and measured FA maps in axial (**a**–**h**), coronal (**i**–**p**) and sagittal (**q**–**y**) central slices within the pig body phantom with zero-phases, HW phases and after static phase ${B}_{1}^{+}$ shimming with PV1 and with PV2.

Figure 3 demonstrates the results of the simulated combined ${B}_{1}^{+}$ field distribution and the experimentally measured FA maps in transversal, coronal, and sagittal central slices within the pig body phantom before optimization (with zero-phases), HW phases, and after ${B}_{1}^{+}$ shimming (with two optimized phase vectors PV1 and PV2). It can be seen in the EM simulation results that the implementation of the HW phases led to an enhancement in ${{\rm{Tx}}}_{{\rm{eff}}}$ by factor > 3 (from 2.2 to 6.9 $\mu {\rm{T}}/\sqrt{{\rm{kW}}}$), without any additional RF-shimming compared to zero-phases. This proves that the initially optimized HW phases were close to optimal regarding maximization of the transmit efficiency. With PV1, the RSD was decreased by factor > 3 (from 0.35 to 0.10) and ${{\rm{Tx}}}_{{\rm{eff}}}$ was increased by factor > 3 (from 2.2 to 6.7 $\,\mu {\rm{T}}/\sqrt{{\rm{kW}}}$) compared to zero-phases. With PV2, the RSD was decreased by factor > 2 (from 0.35 to 0.15) and the ${{\rm{Tx}}}_{{\rm{eff}}}$ was increased by factor > 3.4 (from 2.2 to 7.5 $\,\mu {\rm{T}}/\sqrt{{\rm{kW}}}$) compared to zero-phases. With HW phases and even with prior pTx shimming, the mean FA was increased by 80% (from 10° to 18°) compared to zero-phases [see Fig. 3(f)]. The RSD computed in the selected ROI of the measured FA map was improved by factor > 3 (from 0.32 to 0.10) compared to zero-phases. It is interesting to see that with PV2, the RSD was improved by factor > 6 and factor > 2 compared to zero-phases and HW phases, respectively [see Fig. 3(h)]. The enhancement in the ${B}_{1}^{+}$ field penetration for PV2 compared to zero-phases, HW phases and PV1 is particularly clear in the simulated and measured central coronal [see Fig. 3(i–p)] and sagittal [see Fig. 3(q–y)] slices.

**Figure 4:**
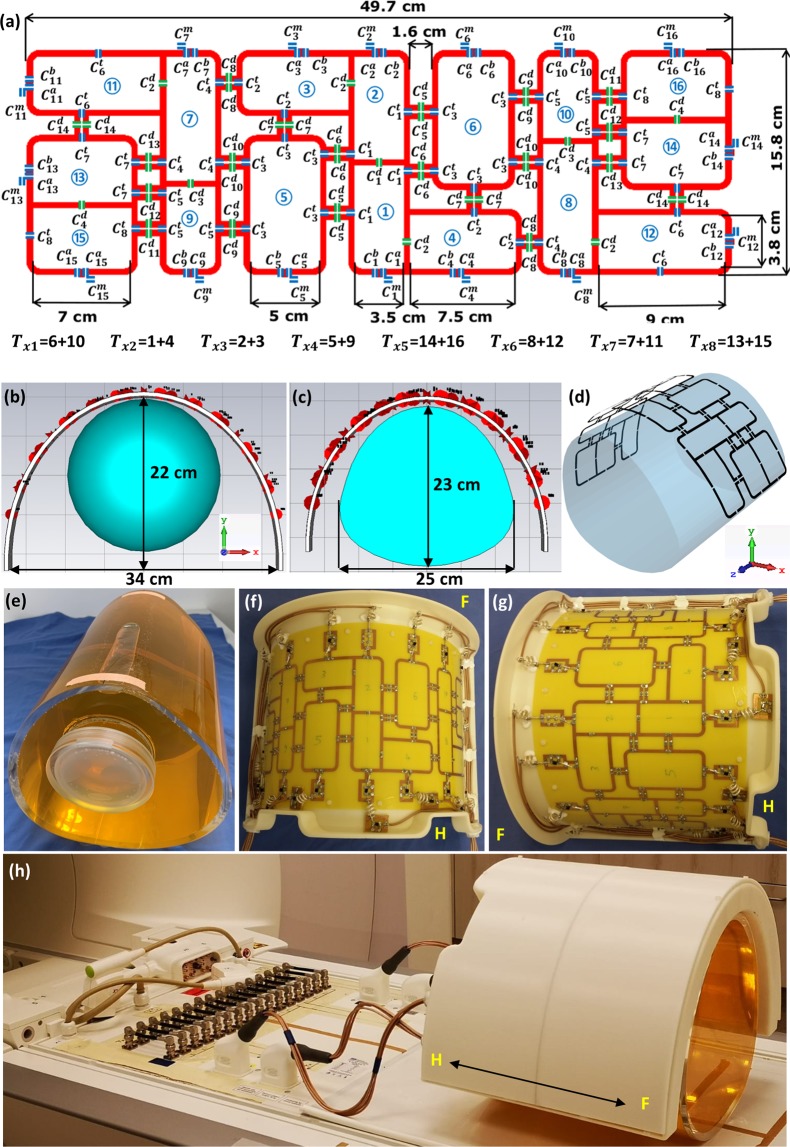
Schematic of the mono-surface array with element dimensions, capacitor variables, and channel numbers (${T}_{x1}$**–**${T}_{x8}$) (**a**), RF coil simulation model loaded with a 20 cm diameter spherical phantom (**b**) and a pig body phantom (**c**,**d**). (**e**) Prototype of the in-house developed pig body phantom. Prototype of the mono-surface array shown from the head−foot (**H**–**F**) direction (**f**) and side view (**g**). (**h**) The mono-surface array setup loaded with a pig body phantom and connected to the interface via four ODU plugs and to the 7 T scanner.

Good agreement between the simulated combined transversal ${B}_{1}^{+}$ field distributions and the measured FA maps was achieved for zero-phases, HW phases and PV2. However, there were some discrepancies between the CST simulation results and the experimentally measured FA maps for PV1. The potential sources of observed discrepancies are: (i) light asymmetry of the pig phantom prototype compared to the ideal simulation model [see Fig. 4(c,e)] and (ii) shift in phantom position with respect to the coil in comparison to simulated position.

**Figure 5:**
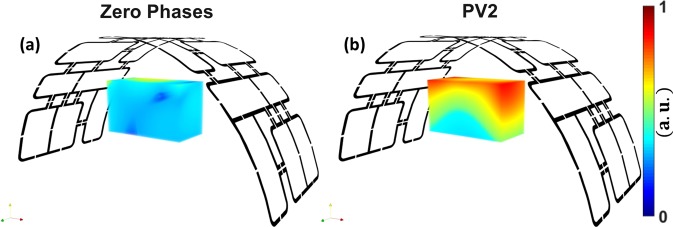
Simulated 3D SNR improvement achieved by the static ${B}_{1}^{+}$ shimming with PV2 compared to zero-phases.

Figure 5 shows the simulated improvement of the SNR achieved by static ${B}_{1}^{+}$ field shimming of the selected phase vector PV2 obtained using our in-house developed phase optimization method. An increase in mean SNR (a.u.) was observed in the whole volume of the pig body phantom from 36 before shimming with zero-phases to 83 after shimming with PV2 (i.e., factor 2.3), in median value of SNR within phantom from 14 before shimming to 25 after shimming with PV2 (i.e., factor 1.7), and in volume within SNR is 83 iso-surface (i.e., “SNR above mean for optimized case”): by a factor of 1.7 and an absolute volume increase of 1362 cm^3^.

### ${{\boldsymbol{B}}}_{1}^{+}$ shimming using the pTx system

#### Combined FA maps

**Figure 6:**
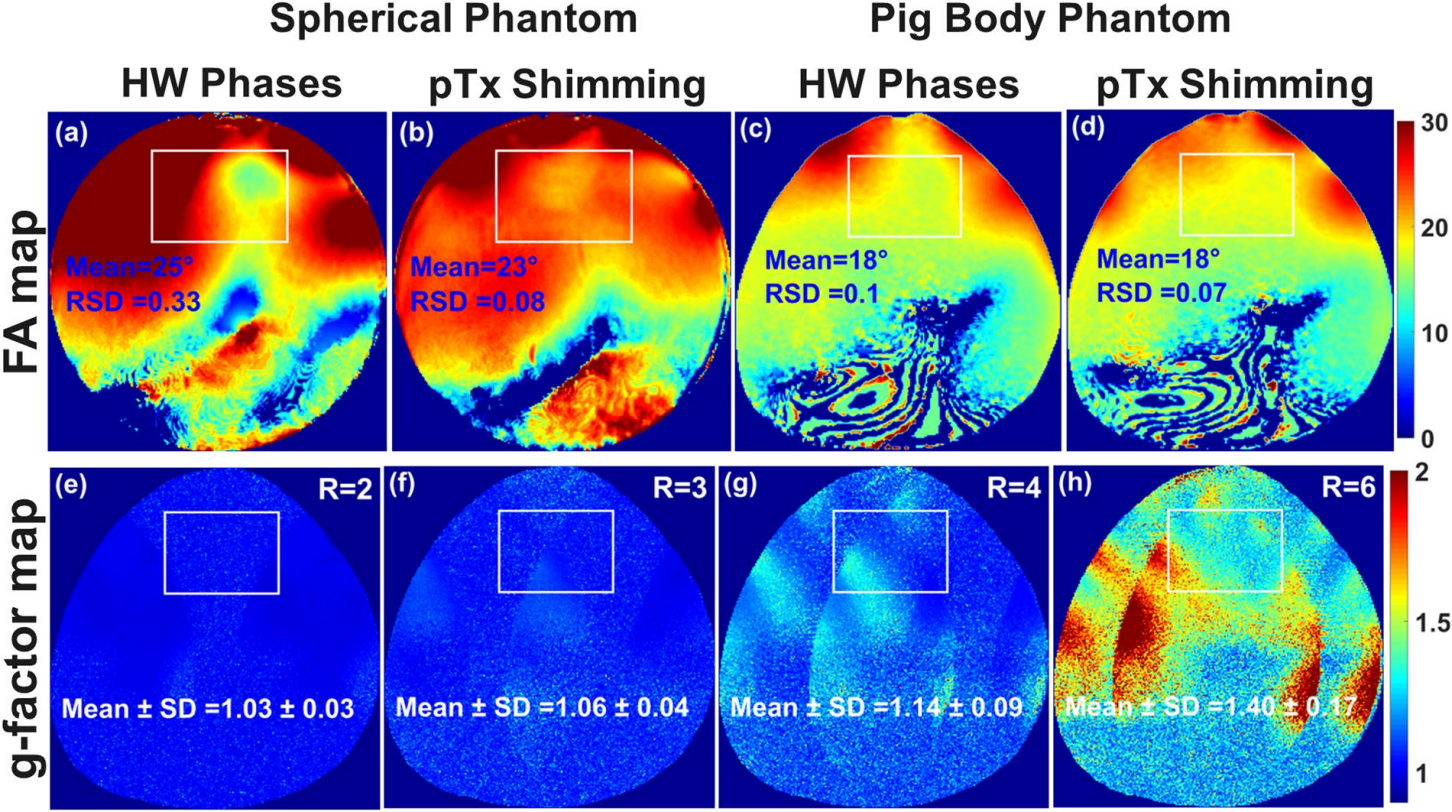
Measured central transversal FA maps in degrees acquired using the mono-surface array within a spherical phantom of 20 cm in diameter (**a**,**b**) and within a pig body phantom (**c**,**d**) before on-scanner static ${B}_{1}^{+}$ shimming using the vendor shimming algorithm. (**e**–**h**) Measured g-factor maps within the pig body phantom with acceleration in the L−R direction for R = 2, 3, 4, and 6, respectively. The mean ± SD of the g-factor values were computed in the selected ROI.

Figure 6(a–d) illustrates the measured combined central transversal FA maps in the 20 cm diameter spherical phantom and the pig body phantom acquired using the mono-surface array without shimming and after on-scanner static ${B}_{1}^{+}$ shimming with both amplitude/phases using the vendor integrated pTx shimming algorithm. It is important to notice that the found amplitudes for the pig body phantom were approximately equal for all 8Tx-channels [see Supplementary Table S3], which means that static ${B}_{1}^{+}$ shimming was performed with the maximum amplitude efficiency. For the spherical phantom [see Fig. 6(a,b)], the on-scanner ${B}_{1}^{+}$ shimming improved the FA homogeneity coefficient by factor > 4 (the RSD was decreased from 0.33 to 0.08) in the selected 3D ROI (10 × 10 × 1.0 cm^3^) compared to before shimming with HW phases. For the pig body phantom, the RSD has improved from 0.10 before pTx shimming to 0.07 after pTx shimming. This corresponds to a 43% improvement in the FA homogeneity coefficient compared to before shimming with HW phases. The vendor integrated static ${B}_{1}^{+}$ shimming in combination with the mono-surface array allows for shaping a relatively homogeneous profile of the FA in the area of the heart within the pig body phantom [see Fig. 6(c,d)].

#### g-factor maps

Figure 6(e–h) demonstrates the measured g-factor maps acquired using the mono-surface array with acceleration factors of R = 2, 3, 4, and 6 after a vendor integrated on-scanner pTx ${B}_{1}^{+}$ shimming. For accelerations defined in the left−right (L−R) direction, the g-factor in the region corresponding to the position of the pig’s heart increases gradually with acceleration factors. The mean values of g-factor for accelerations from R = 2 to 6 changes from 1.03 to 1.40, respectively, being very moderate values for a typical cardiac array. That way, an acceleration up to R = 6 is possible in L−R direction whereas for classical double-surface arrays with individual anterior and posterior parts, the acceleration regime is practically not feasible with R > 3.

### MRI measurements with pig cadavers

#### Cadaver #1 (68 kg)

**Figure 7:**
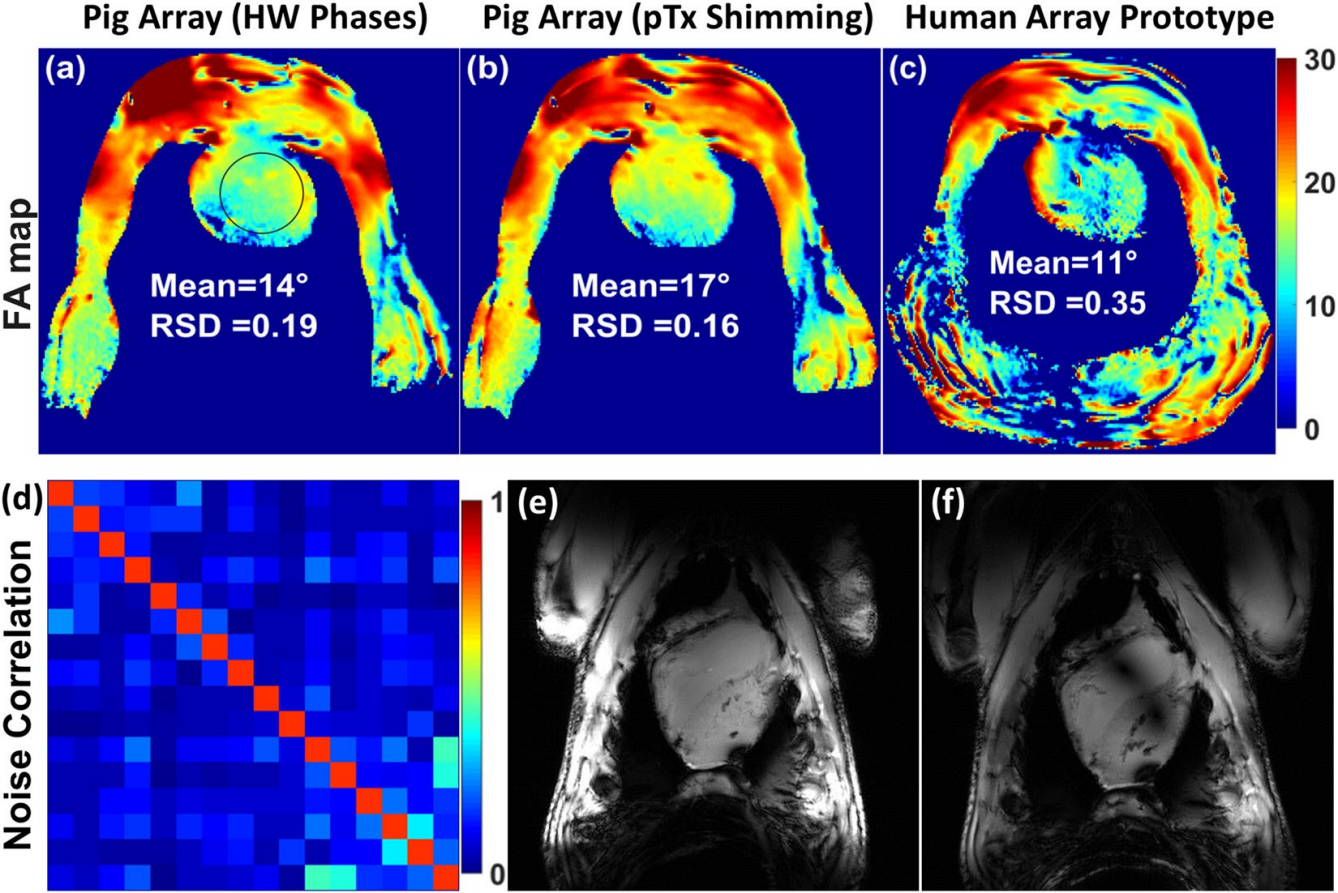
Measured FA maps in transversal view acquired using the mono-surface array within a cadaver pig #1 (68 kg) without on-scanner shimming (with HW phases) (**a**), and after on-scanner static ${B}_{1}^{+}$ shimming using the vendor shimming algorithm (**b**). (**c**) Transversal FA map acquired from the same animal using an 8Tx/16Rx human cardiac array prototype. (**d**) Measured noise correlation matrix from the mono-surface array loaded with a pig cadaver. (**e**,**f**) Coronal views demonstrate relatively homogeneous ${B}_{1}^{+}$ field excitation using the dedicated mono-surface array (**e**) while the coronal images acquired using the human cardiac array prototype shows clearly visible destructive ${B}_{1}^{+}$ artefacts (**f**).

Figure 7(a–c) illustrate the combined FA maps in short axis (SA) views within the whole thorax of pig cadaver #1 (68 kg) acquired using the mono-surface array without shimming and after on-scanner pTx ${B}_{1}^{+}$ shimming compared to the human array prototype. The coil showed good homogeneity in the FA map with a computed FA mean value of 14° before pTx shimming. After the vendor’s ${B}_{1}^{+}$ shimming, the mean FA was increased by about 21%. With the pig cadaver #1, the vendor static ${B}_{1}^{+}$ shimming has improved the RSD from 0.19 to 0.16. For the recently published^44^ antisymmetric anterior coil array comprising of 8-elements combined with a rectilinear 8-elements posterior array and optimized for smaller pigs (<50 kg), the RSD was computed as 0.46 within the pig heart. This result shows that static ${B}_{1}^{+}$ shimming using pTX RFPA combined with the mono-surface array significantly improved the ${B}_{1}^{+}$ field homogeneity coefficient (factor > 2.8) compared to an 8Tx/16Rx pig cardiac array comprising two independent A−P parts. The RSD in the pig cadaver #1 using the mono-surface array was improved by factor > 2 (from 0.35 to 0.16) compared to the human array prototype [see Fig. 7(b,c)].

Figure 7(d) shows the noise correlation matrix acquired using the mono-surface array loaded with the pig cadaver of 46 kg. Despite the complexity of the mono-surface array, the maximum noise correlation was 0.4. Figure 7(e,f) depicts coronal views demonstrating a relatively homogeneous ${B}_{1}^{+}$ field excitation acquired using the mono-surface array, while destructive ${B}_{1}^{+}$ artefacts are clearly visible for the human array prototype.

**Figure 8:**
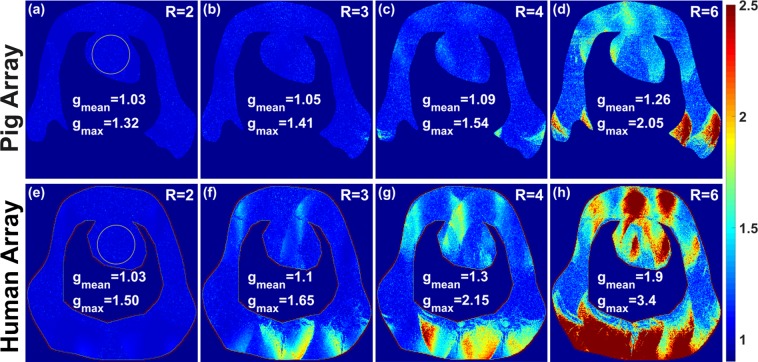
Measured g-factor maps of R = 2, 3, 4, and 6 within a cadaver pig #1 (68 kg) with acceleration in the L−R direction were acquired using the mono-surface array (**a**–**d**) compared to an 8Tx/16Rx human cardiac array prototype (**e**–**h**). The mean and maximum of the g-factors were evaluated in the ROI of the heart. The mono-surface array geometry and housing shaping provide clearly observed benefit for the parallel imaging acceleration providing up to 50% lower g-factor for the high acceleration rates (R = 6) compared to the human cardiac array prototype.

Figure 8 demonstrates the parallel imaging performance of the mono-surface array compared to the human cardiac array prototype. G-factor maps were acquired for acceleration factors of R = 2, 3, 4, and 6 in a pig cadaver #1 (68 kg). For both coils, the acceleration was set in the L−R direction. For the mono-surface array, the mean of the g-factor in the heart ROI was evaluated as: 1.03, 1.05, 1.09, and 1.26 for R = 2, 3, 4 and 6, respectively. It is important to notice that the dedicated mono-surface array provides improved parallel imaging capabilities with up to 50% lower g-factor for the high acceleration rates (R = 6) compared to human array prototype.

#### Cadaver #2 (46 kg)

**Figure 9:**
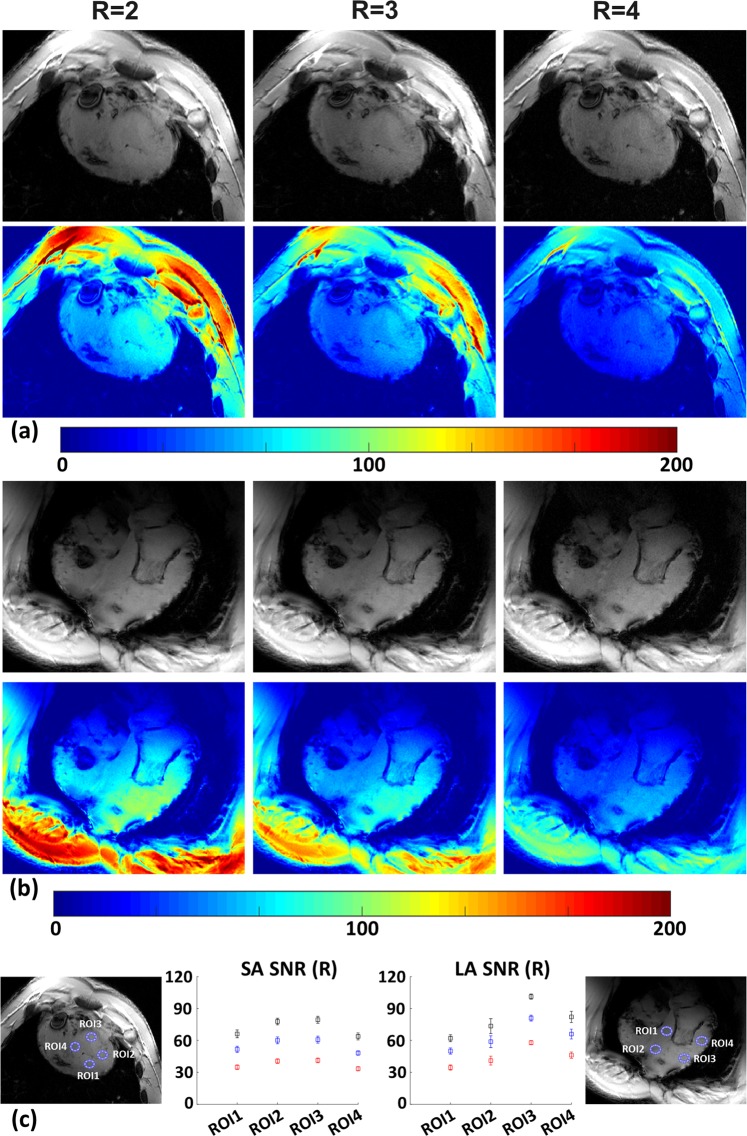
Impact of parallel imaging acceleration on SNR in anatomical images within a cadaver pig #2 (46 kg). (**a**) Anatomical SA and corresponding SNR maps for varying acceleration factors R = 2, 3 and 4 (left to right). (**b**) Respective LA views analogous to (**a**). (**c**) SNR plots of mean ± single standard deviation in four myocardial ROIs for SA and LA views in dependence of the acceleration factor. Black: R = 2, Blue: R = 3, and Red: R = 4.

Figure 9 shows SA [see Fig. 9(a)] and long axis (LA) [see Fig. 9(b)] views acquired using R = 2, 3 and 4 in a pig cadaver #2 (46 kg). Images in the second row show SNR maps for the respective anatomical images. Placement of the four LV ROIs and the respective SNR in dependence of the acceleration factor are displayed in Fig. 9(c). Mean SNR values in the ROIs range from 64–79, 48–61, 33–41 (SA view) and 62–101, 50–81, 34–58 (LA view) for acceleration factors R = 2, 3, 4, respectively. For the SA view this corresponds to a drop of about 23% and 48% for R = 3 and R = 4. Respective drops for the LA view are about 20% and 44% for R = 3 and R = 4.

### *In-vivo* MRI measurements with pig (60 kg)

**Figure 10:**
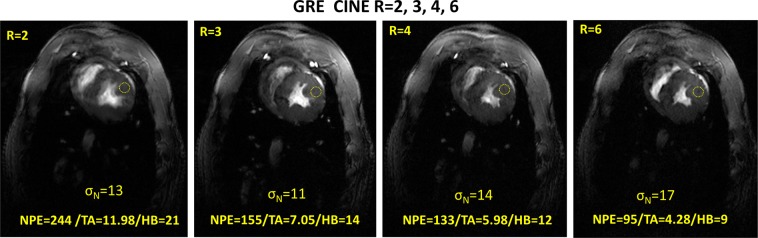
Single frames of the GRE CINE images of the pig heart acquired in a 60 kg pig *in-vivo* with parallel imaging acceleration factors R = 2, 3, 4, and 6. Spatial resolution 0.6 mm × 0.6 mm × 6 mm, FA = 35°, retrospective reconstruction 30 phases, frame at trigger delay = 270 ms. For the large number of PE-lines significant blurring of the heart is observed due to relatively large number of HB needed for acquisition and probably poor consistency of the acquired k-space. This effect is removed by higher acceleration (less PE-steps) and, thus, less amount of HB needed. With R = 3 and 4 the myocardium wall has clear sharp contours.

Figure 10 shows *in-vivo* single frames of the GRE CINE images of the pig heart acquired with parallel imaging acceleration factors R = 2, 3, 4 and 6. The number of phase encoding steps (NPE), TA, and number of heart beats (HB) used for acquisition are shown in the corresponding images. For the large number of PE-lines at R = 2 a significant blurring of the heart wall is observed. This is due to the large amount of HB required during acquisition and, which often results in poor consistency of the acquired image k-spaces due to intrinsic variation of the both heart rhythm and motion. This effect is removed by higher acceleration (less PE-steps), where, a lower amount of HB is needed. With the acceleration factors R = 2, 3, 4 and 6, visualization shows clear sharp contours for the myocardium wall.

**Figure 11:**
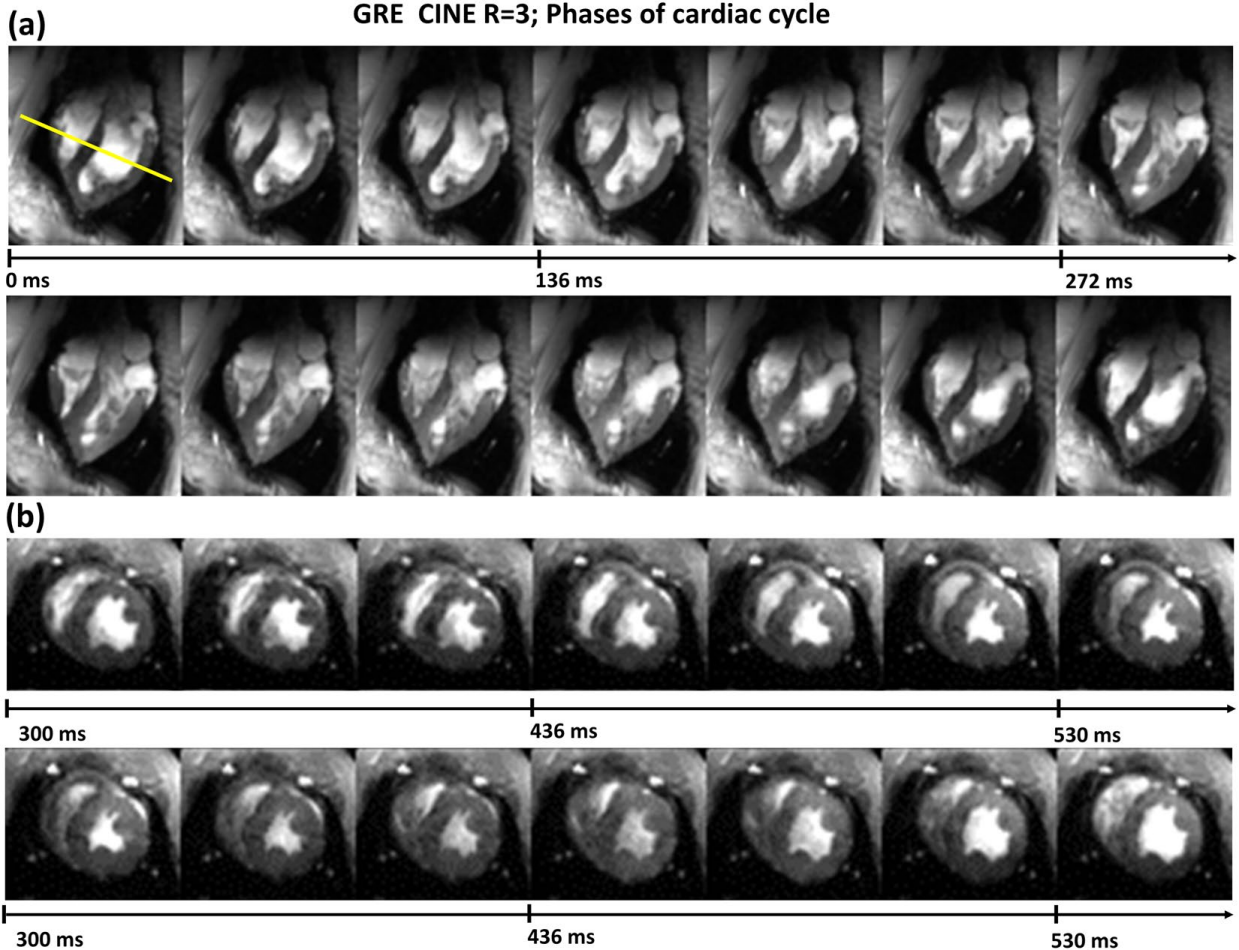
Multiple heart phases acquired in a 60 kg pig *in-vivo* with GRE CINE at acceleration factor R = 3. LA view (**a**) and SA view (**b**) are shown. High spatial resolution of 0.6 mm in-plane is demonstrated while keeping fair diagnostic quality SNR. This is essentially superior to the typical clinical cMRI at 3 T which usually is obtained at in-plane spatial resolutions of 1–1.5 mm.

Figure 11 show *in-vivo* multiple heart phases acquired with GRE CINE at acceleration factor R = 3 (every second reconstructed heart phase is shown).

## Discussion

In this study we designed, simulated, built, and tested a mono-surface 8Tx/16Rx pTx coil array for cMRI in pigs at 7 T. The mono-surface array was driven in pTx mode on a 7 T scanner. The mono-surface array shows a significant improvement in the ${B}_{1}^{+}$ field homogeneity after RF-shimming, which proves the high efficiency of the developed array design for using with the pTx-capable MR-system in large animal cardiac studies at UHF.

The PCB was fixed on one half-elliptical housing, which was designed to fit pigs ranging from 40–80 kg [see Fig. 4(f–h)]. The mono-surface array enabled efficient ${B}_{1}^{+}$ shimming capabilities and showed better control of the ${B}_{1}^{+}$ field distribution for all individual 8Tx channels in a spherical phantom [see Fig. 1] and in a pig body phantom [see Fig. 2]. However, both simulated and measured ${B}_{1}^{+}$ field distribution per channel within a 20 cm diameter spherical phantom shows some stronger overlaps between the channels ${T}_{x2}$ (combined elements 1 & 4), ${T}_{x3}$ (combined elements 2 & 3) and ${T}_{x4}$ (combined elements 5 & 9) [see Fig. 1(b,c,d,f,g,h)]. This overlap of the ${B}_{1}^{+}$ profiles in the central coronal plane at about 10 cm depth from the coil surface has many reasons. First, the spherical phantom was selected to test the worst-case scenario for loading conditions, where the side elements are not loaded properly (e.g., ${T}_{x5}$ for combined elements 14 & 16, and ${T}_{x6}$ for combined elements 8 & 12) and similarly for the antisymmetric channels ${T}_{x7}\,\,$and ${T}_{x8}$. For example, based on bench-top S-parameter measurements for the individual 16 elements, the channels ${T}_{x7}$ (combined elements 7 & 11) and ${T}_{x8}$ (combined elements 13 & 15) present poor decoupling (e.g., ${{\rm{S}}}_{13,15}\,$ =  −8 dB and ${{\rm{S}}}_{14,16}\,$ =  −9 dB) [see Supplementary Table S1]. Second, there is ± 10° error in the measured relative HW phase per element, which was measured at distance of about 10 cm from the element surface. The measured relative HW phases were used to compute the required RF cable phase shifters, which were used to implement the optimal phase vectors PV1 and PV2 [see Table 2]. However, the mono-surface array demonstrated accepted decoupling when loaded with the pig body phantom (${{\rm{S}}}_{13,15}\,$ =  −11 dB and ${{\rm{S}}}_{14,16}\,$ =  −10 dB) [see Table 1] and with a 46 kg pig cadaver (${{\rm{S}}}_{13,15}\,$ =  −16 dB and ${{\rm{S}}}_{14,16}\,$ =  −12 dB) [see Supplementary Table S2].

**Table 2:** Measured relative HW phases for the mono-surface array loaded with the pig body phantom, relative optimized phases (PV1 and PV2), and the required coaxial cable PSs required to validate the optimal vectors PV1 and PV2. The coaxial cable PSs were computed after subtracting the HW phases from PV1 and PV2, subtraction the results from the maximum positive phase value and finally rounding the resulted phase nearest to 22.5°.

pTx Channel #	Element#	HW Phases[°]	PV1[°]	PV2[°]	Cable PSs for PV1 [°]	Cable PSs for PV2 [°]
${{T}}_{{x}1}$	6	−98	−25	59	45	90 + 22.5
	10	−91	4.0	48	22.5	90 + 45
${{T}}_{{x}2}$	1	−30	81	114	0	90 + 22.5
	4	15	12	11	90 + 22.5	180 + 90
${{T}}_{{x}3}$	2	−66	19	91	22.5	90 + 22.5
	3	−113	−246	148	180 + 45 + 22.5	0
${{T}}_{{x}4}$	5	13	110	181	22.5	90
	9	70	−214	−71	45	45
${{T}}_{{x}5}$	14	87	−111	−4.0	180 + 90 + 45	0
	16	82	−169	−77	0	45 + 22.5
${{T}}_{{x}6}$	8	136	−47	3.0	180 + 90 + 22.5	45
	12	164	−106	−72	22.5	90 + 45
${{T}}_{{x}7}$	7	68	−188	−139	0	90 + 22.5
	11	98	−96	247	180 + 90 + 45	90 + 22.5
${{T}}_{{x}8}$	13	140	−164	−67	45	90 + 22.5
	15	164	−82	−35	0	90

The capacitor values optimized on bench-top measurements were in good agreement with those capacitor values initially optimized in CST-DS (pig body phantom as load) [see Table 3]. For some elements, there are discrepancies between the optimized capacitor values in CST-DS simulations and on bench-top measurements, which mainly arises at the feeding ports of the splitting tuning and matching capacitors (e.g., ${C}_{14}^{m}$, ${C}_{14}^{a}$ and ${C}_{14}^{b}$ for element 14). This is because the discrete implemented PS circuits were added to adjust the HW phases, and they were not modeled in the CST simulations since they were considered to be ideal 50 Ω circuits. However, in the real coil prototype, the additional discrete PSs might change the input impedance at the coil feeding ports, which cause some discrepancies among capacitors optimized using simulations and bench-top measurements.

**Table 3:** Optimized capacitor values of the mono-surface array in pF obtained using CST RF-circuit co-simulation loaded with the dedicated pig body phantom and final optimized capacitor obtained using bench-top measurements loaded with the pig cadaver of 46 kg.

Capacitor	Bench	Simulation	Capacitor	Bench	Simulation
${C}_{1}^{d}$	12.7	12.7	${C}_{14}^{m}$	10.0	6.20
${C}_{2}^{d}$	12.7	12.7	${C}_{15}^{m}$	8.20	6.20
${C}_{3}^{d}$	13.3	13.3	${C}_{16}^{m}$	8.20	6.20
${C}_{4}^{d}$	7.20	7.20	${C}_{1}^{a}$	10.0	8.20
${C}_{5}^{d}$	10.0	10.0	${{\boldsymbol{C}}}_{2}^{{\boldsymbol{a}}}$	10.0	8.20
${C}_{6}^{d}$	0.50	0.50	${C}_{3}^{a}$	6.20	6.20
${C}_{7}^{d}$	1.00	1.00	${C}_{4}^{a}$	6.20	6.20
${C}_{8}^{d}$	1.00	1.00	${C}_{5}^{a}$	4.70	2.10
${C}_{9}^{d}$	10.0	10.0	${C}_{6}^{a}$	4.70	2.10
${C}_{10}^{d}$	1.00	1.00	${C}_{7}^{a}$	6.20	6.20
${C}_{11}^{d}$	1.00	1.00	${C}_{8}^{a}$	8.20	6.20
${C}_{12}^{d}$	1.00	1.00	${C}_{9}^{a}$	10.0	8.20
${C}_{13}^{d}$	1.00	1.00	${C}_{10}^{a}$	8.20	8.20
${C}_{14}^{d}$	2.10	2.10	${C}_{11}^{a}$	6.20	6.20
${C}_{1}^{t}$	7.20	7.20	${C}_{12}^{a}$	8.20	6.20
${C}_{2}^{t}$	6.20	6.20	${C}_{13}^{a}$	8.20	8.20
${C}_{3}^{t}$	8.20	8.20	${C}_{14}^{a}$	10.5	8.20
${C}_{4}^{t}$	10.0	10.0	${C}_{15}^{a}$	10.0	10.0
${C}_{5}^{t}$	12.1	12.1	${C}_{16}^{a}$	10.0	10.0
${C}_{6}^{t}$	4.70	4.70	${C}_{1}^{b}$	11.6	10.0
${C}_{7}^{t}$	10.0	10.0	${C}_{2}^{b}$	11.0	10.0
${C}_{8}^{t}$	6.70	6.70	${C}_{3}^{b}$	9.20	8.20
${C}_{1}^{m}$	6.20	6.20	${C}_{4}^{b}$	10.9	8.20
${C}_{2}^{m}$	6.20	6.20	${C}_{5}^{b}$	6.70	6.20
${C}_{3}^{m}$	6.20	6.20	${C}_{6}^{b}$	4.70	6.20
${C}_{4}^{m}$	4.70	6.20	${C}_{7}^{b}$	6.20	7.80
${C}_{5}^{m}$	10.0	12.0	${C}_{8}^{b}$	8.20	7.80
${C}_{6}^{m}$	8.20	12.0	${C}_{9}^{b}$	8.20	9.00
${C}_{7}^{m}$	10.0	10.0	${C}_{10}^{b}$	9.20	9.00
${C}_{8}^{m}$	10.0	10.0	${C}_{11}^{b}$	8.20	7.40
${C}_{9}^{m}$	10.0	10.0	${C}_{12}^{b}$	6.20	7.40
${C}_{10}^{m}$	10.0	10.0	${C}_{13}^{b}$	13.3	10.0
${C}_{11}^{m}$	10.0	4.70	${C}_{14}^{b}$	8.20	10.0
${C}_{12}^{m}$	10.0	4.70	${C}_{15}^{b}$	12.7	10.0
${C}_{13}^{m}$	10.0	6.20	${C}_{16}^{b}$	10.0	10.0

The developed 8Tx/16Rx cardiac array demonstrated high efficiency in both Tx and Rx properties for cMRI at 7 T. Due to the good decoupling and low correlation between the ${B}_{1}^{+}$ fields generated by all individual 16 elements it enables efficient capabilities for static phase ${B}_{1}^{+}$ shimming using phases control [see Fig. 3] and pTx RFPA based ${B}_{1}^{+}$ shimming with both amplitudes/phases in phantom [see Fig. 6] and pig cadaver [see Fig. 7] providing improved homogeneity and penetration of the ${B}_{1}^{+}$ field. A comparison between an antisymmetric coil array and a standard rectilinear symmetric coil array has previously been made^44^. The main issue of the standard rectilinear coil array design is the coupling between the diagonal elements. The coupling between neighboring and diagonal elements can be minimized by the optimal distribution of the coil elements of the array. The basic idea of the mono-surface array design is to move some elements from the longitudinal distribution in the *z*-axis in a standard rectilinear design^44^ to the transversal direction in the *x*-axis [see Fig. 4(a)]. Then, the central four elements (e.g., elements 1–4) together form two antisymmetric L-shaped channel pairs driven by independent two RFPA. This will facilitate the decoupling of the central four elements by using a common conductor and a shared decoupling capacitor^30,31,43,44,53^ (SDC) (i.e., between elements 1 & 2, elements 2 & 3, and elements 1 & 4). Additionally, this gives more degrees of freedom to distribute the surrounding two elements 5 and 6 via the selection of five different decoupling locations using capacitive decoupling mechanism (${C}_{5}^{d}$, ${C}_{6}^{d}$, ${C}_{7}^{d}$, ${C}_{9}^{d}$, and ${C}_{10}^{d}$) in conjunction with a gap of 1.5 cm from each side of the array. The dimensions of the antisymmetric elements were selected to create a balance between the optimal dimensions of the individual 16-loops and the total external dimensions of the array to fit perfectly with the half-elliptical housing. Additionally, instead of extending the other loop elements in the longitudinal *z*-axis like in the standard rectilinear array design, eight elements (3, 4, 11, 12, 13, 14, 15 and 16) were extended in the transversal *x*-axis. These elements results in a stronger penetration of the ${B}_{1}^{+}$ field within the pig heart after bending.

The phase vector optimization results reveal the significant advantage of the mono-surface array in terms of static phase ${B}_{1}^{+}$ shimming optimization potential. The new mono-surface array design provides additional degrees of freedom for ${B}_{1}^{+}$ shimming (e.g., the L-shaped element distribution, loop sizes and geometries, and the antisymmetric 16 elements allocation on the half-elliptical housing). Even without RFPA-based static ${B}_{1}^{+}$ shimming, the developed coil array with the optimized HW phases showed homogeneous FA map distribution (RSD of 0.19) without noticeable destructive interferences within the heart region [see Fig. 7(a)]. After on-scanner RFPA-based static pTx ${B}_{1}^{+}$ shimming with the RSD of the measured FA within the heart region of pig cadaver #1 is 0.16 [see Fig. 7(b)]. This result shows that the mono-surface array after on-scanner RFPA-based static pTx ${B}_{1}^{+}$ shimming has a significant improvement in the ${B}_{1}^{+}$ field homogeneity becoming by factor of 2.8 higher compared to a pTx cardiac array of similar design with two independent A−P parts (RSD = 0.46)^44^.

Due to the difficultly to find in literature a dedicated multichannel pTx coil array for pigs at 7 T, we compared our new array design to some published human arrays. The measured mean g-factors that we report in this work using the dedicated coil array in a pig cadaver #1 with acceleration factors of R = 4 (1.09) [see Fig. 8(a–d)] is lower than the mean g-factors mentioned in the literature (1.58, 2.33, and 1.2) using 8-channel^30^, 16-channel^31^ and 32-channel^26^ Tx/Rx loop human coil arrays, respectively. The mean g-factor obtained from the mono-surface array with acceleration factors of R = 6 is 1.26, which is about 58% lower than the measured mean g-factor mentioned in the literature^26^ (${{\rm{g}}}_{{\rm{mean}}}$  =  2) with 32-channel Tx/Rx human coil array. High resolutions (0.3 mm × 0.3 mm) cardiac images were acquired using the mono-surface array in a pig cadaver #2 [see Fig. 9]. The mono-surface array provides relatively small intensity variation across the region of the heart. In comparison to the smaller designs with two independent arrays^43,44^, the mono-surface array reveals practically negligible noise amplification over R = 2, 3, and 4. The posterior wall in a SA view remains clearly visible for acceleration factors R = 2, 3, and 4, indicating that the coil is capable for high quality volumetric cMRI acquisitions in *in-vivo* use.

The demonstrated *in-vivo* results confirm that the optimized phase vector (PV2) provides homogeneous ${B}_{1}^{+}$ excitation of the heart region without visible destructive interference and sufficiently good coverage of the posterior rear wall [see Fig. 10]. The noise level (${{\rm{\sigma }}}_{{\rm{N}}}$) computed as standard deviation in marked ROIs increases with decreasing number of PE-lines. An average tissue-to-blood contrast of eight is observed on both LA and SA views. The mono-surface array has high parallel imaging capability, enabling high spatial resolution cMRI. The antisymmetric design allows for efficient usage of the acceleration with phase-encoding direction angulated according to the standard anatomical views of the heart. A low acceleration factor (R = 2) resulted in blurring of the *in-vivo* images due to the relatively large amount of PE-lines and thus, HB used for CINE reconstruction. This was, however, efficiently handled by increasing the acceleration and correspondingly decrease the number of PE-lines needed for the reconstruction. By this way, errors originated from losing k-space coherence were effectively prevented. No visible artefacts of GRAPPA reconstruction are observed in the *in-vivo* scans despite the significant reduction of total PE-steps. Both LA and SA anatomical views provided high spatial (0.6 mm in-plane) and sufficient temporal resolution while keeping fair diagnostic quality SNR [see Fig. 11]. The *in-vivo* measurements using the mono-surface array confirmed high potential of the antisymmetric design in terms of both transmit and receive properties. Both LA and SA views could be visualized with high spatial resolution if a drastically reduced number of phase encoding steps (and number of heart cycles) was used for the reconstruction of 30 cardiac phases. The spatial resolution of 0.6 mm achieved *in-vivo* is essentially superior to that used under typical clinical cMRI conditions at 3 T (1–1.5 mm in-plane spatial resolution).

The mono-surface antisymmetric array design, hardware electronics, RF shimming simulations described in this paper can be easily adapted for multichannel coil arrays optimized for different MR applications (e.g., cardiac, head, and spine) at UHF. The number of loop elements can be easily increased to 32 or even 64, while maintaining good decoupling among all elements.

## Methods

### Array design

The antisymmetric coil array was composed of a mono-surface with 16-elements which were arranged so that the central two elements were arranged anti-symmetrically and flanked by seven elements on either side. Figure 4(a) shows the schematic of the dedicated mono-surface antisymmetric array for pigs. The 14 loop elements were distributed around the central two loop elements 1 and 2 in an antisymmetric arrangement to reduce element coupling due to less overall proximity of neighboring elements^44^. In addition, this method aims to minimize the overlapping of the ${B}_{1}^{+}$ profiles from each individual Tx channel in the heart region of the pig. The dimensions of the central loops 1 and 2 were selected as 3.50 cm × 7.35 cm as described in^43,44^. The decoupling between the central two elements 1 and 2 is accomplished using a common conductor and a SDC^30,31,43,44,53^ of ${C}_{1}^{d}$. The size of the antisymmetric L-shaped elements 3 and 4 was 3.80 cm × 7.50 cm. The antisymmetric L-shaped elements 3 and 4 were decoupled from the loop elements 2 and 1 using a SDC of ${C}_{2}^{d}$. The elements 1, 2, 3 and 4 formed a pair of antisymmetric L-shaped channels (${T}_{x2}$ and ${T}_{x3}$) to reduce coupling between the neighboring elements 5 and 6. The size of the elements 5 and 6 was selected as 5.0 cm × 9.0 cm. Both loop elements have the larger dimensions in the array in order to make a balance between the dimensions of all surrounding loops in the array. This results in five different locations for capacitive decoupling with the surrounding elements (1, 2, 3, 7 and 9) with a gap of 1.5 cm. The elements 1, 2, and 3 were decoupled from the neighboring elements 5 and 6 using capacitive decoupling mechanism of two equal capacitors of ${C}_{5}^{d}$, ${C}_{6}^{d}$ and ${C}_{7}^{d}$ in addition to two gaps of 1.60 cm and 1.1 cm, respectively. The elements 7 and 9 and the antisymmetric identical elements 8 and 10 were decoupled using a SDC of ${C}_{3}^{d}$. Both antisymmetric L-shaped loop elements 11 and 12 were decoupled with elements 7 and 8 using a SDC of ${C}_{2}^{d}$. The elements 13 and 15 and the antisymmetric identical elements 14 and 16 were decoupled using a SDC of ${C}_{4}^{d}$. The total external dimension of the array is 15.8 cm × 49.7 cm.

### EM simulations

EM simulations were performed using CST Microwave Studio (CST-MWS) (Computer Simulation Technology AG Darmstadt, Germany). Figure 4(b–d) illustrates the RF-simulation models of the dedicated cardiac coil array as simulated in CST-MWS. In this paper, the mono-surface array was simulated with two different phantom loads (a 20 cm diameter spherical phantom and a pig body phantom [see Fig. 4(b,c)]. Both phantoms have the same electrical properties with ${{\rm{\varepsilon }}}_{{\rm{r}}}$ = 59.3 and $\sigma$ = 0.79 S/m. All feeding ports, tuning, matching, and decoupling capacitors were modeled as 50 Ω discrete face ports for RF circuit co-simulations to obtain the initial values of capacitors using CST-DS^54^. The array has a copper (Cu) track width of 4 mm and a thickness of 35 *μ*m etched on a 0.3 mm FR4 (${{\rm{\varepsilon }}}_{{\rm{r}}}$ = 4.24 and $\tan \,{\rm{\delta }}$ = 0.014 at 297.2 MHz) PCB (Q-print Electronic GmbH, Heddesheim, Germany). The PCB was bent and fixed around the half-elliptical housing (${x}_{diamter}\,$ = 34 cm and ${y}_{rad}\,$ = 22 cm and 5 mm thickness) made from ABS (${\varepsilon }_{r}$ = 2.8 and $\tan \,\delta$ = 0.0095 at 297.2 MHz) [see Fig. 4(b)]. For matching, tuning, and decoupling, fixed chip nonmagnetic capacitors (Voltronics Corp., Denville, NJ, USA) were used. The total number of mesh cells was 40.195 and 43.332 million for the spherical and pig phantoms, respectively. Individual 16-AC simulation tasks corresponding to the 16-elements of the array were evaluated at 297.2 MHz for each load. The 3D-data of the $H$-field for each resonance element was exported to MATLAB (MathWorks, Natick, MA, USA) for post-processing.

The two circularly polarized components of the magnetic field with opposite directions of rotation, which represent the transmit field (${B}_{1}^{+}$) and the receive field (${B}_{1}^{-}$) are given by: ${B}_{1}^{+}=({B}_{x}+i{B}_{y})/2$ and ${B}_{1}^{-}={({B}_{x}-i{B}_{y})}^{\ast }/2$, respectively, where ${B}_{x}$ and ${B}_{y}$ are the complex transversal components of the magnetic field in the $x$ and $y$ directions, $i$ is the imaginary unit, and the asterisk indicates the complex conjugate. The ${\rm{RSD}}={\rm{SD}}({B}_{1}^{+})/\overline{{B}_{1}^{+}}$, and ${{\rm{Tx}}}_{{\rm{eff}}}=\overline{{B}_{1}^{+}}/\sqrt{{P}_{in}}$ in $\mu {\rm{T}}/\sqrt{{\rm{kW}}}$, were evaluated in the selected ROI, where SD is the standard deviation, $\overline{{B}_{1}^{+}}$ is the mean value of the ${B}_{1}^{+}$ field, and ${P}_{in}$ is the stimulated input power in (kW). The mono-surface array was fed with the same total input stimulated power (${P}_{in}$ = 8 W) and all coil elements were fed with equal amplitudes.

The simulated 3D-SNR map within the pig body phantom [see Fig. 5(q,r)] was computed using magnitudes of the combined ${B}_{1}^{+}$ field and ${B}_{1}^{-}$ fields of individual 16-elements, with a low FA (<10°) approximation:

1 $${\rm{SNR}}\propto \sqrt{\mathop{\sum }\limits_{{\rm{k}}=1}^{{\rm{N}}}{({B}_{1}^{+}\cdot {B}_{1,{\rm{k}}}^{-\ast })}^{2}}$$

where the combined ${B}_{1}^{+}$ was given by

2 $${B}_{1}^{+}=\mathop{\sum }\limits_{{\rm{k}}=1}^{{\rm{N}}}{{\rm{b}}}_{1,{\rm{k}}}^{+}({{\rm{\varphi }}}_{{\rm{k}}})$$

where ${{\rm{b}}}_{1{\rm{k}}}^{+}=|{{\rm{b}}}_{1{\rm{k}}}^{+}|{{\rm{e}}}^{{i{\rm{\varphi }}}_{{\rm{k}}}}$ is the complex ${B}_{1}^{+}$ field for each coil element (N = 1–16) and *i* is the imaginary unit.

### Static phase ${{\boldsymbol{B}}}_{1}^{+}$ shimming

To achieve the best possible image quality with the proposed mono-surface array for *in-vivo* MRI studies, comprehensive ${B}_{1}^{+}$ field optimizations of static phase ${B}_{1}^{+}$ shimming within a pig body phantom was performed. Static phase ${B}_{1}^{+}$ shimming was carried out within the pig body phantom using two optimization cost functions within a 3D ROI of 12 × 10 × 1.0 cm^3^ resulting in two optimal phase vectors (PV1 and PV2). The first optimal phase vector (PV1) was found using the optimization of the cost function $\,{F}_{c1}$ of Eq. (3) for maximum ${B}_{1}^{+}$ field homogeneity. The second optimal phase vector (PV2) was found using weighted combination of both ${B}_{1}^{+}$ field homogeneity and transmit efficiency using the cost functions ${F}_{c3}$ of Eq. (5). The optimization cost functions were given by

3 $${F}_{c1}=\frac{\overline{{B}_{1}^{+}}}{{\rm{RSD}}\lfloor {\rm{\max }}({B}_{1}^{+})-(\overline{{B}_{1}^{+}}\,)\rfloor }\cdot {\rm{TXE}}$$

4 $${F}_{c2}=\frac{{\rm{\min }}({B}_{1}^{+})}{{\rm{RSD}}}\cdot {\rm{TXE}}$$

5 $${F}_{c3}={\rm{w}}{F}_{c1}+\sqrt{(1-{{\rm{w}}}^{2})}{F}_{c2}\,{\rm{where}}\,{\rm{w}}=0.8$$

6 $${\rm{where}}\,{\rm{TXE}}=\frac{|{\sum }_{{\rm{k}}=1}^{{\rm{N}}}{b}_{1,{\rm{k}}}(\{{{\rm{\varphi }}}_{{\rm{k}}}\})|}{{\sum }_{{\rm{k}}=1}^{{\rm{N}}}|{b}_{1,{\rm{k}}}(\{{{\rm{\varphi }}}_{{\rm{k}}}\})|}$$

7 $$\{{{\rm{\varphi }}}_{{\rm{k}}}\}={\rm{argmax}}[{F}_{c(1,3)}(\{{{\rm{\varphi }}}_{{\rm{k}}}\})]$$

### Channel pairing for the 8Tx/16Rx pTx system

The pTx system provides 16 kW Tx power output for eight individual feeding lines (8Tx RFPA channels). Each of these lines is then split equally by a 1:2 wilkinson power splitter, both outputs of the power divider fed two elements of the array. Therefore, each of the 8Tx channels was driven by a 2 kW/channel. To form an 8Tx/16Rx coil array, each two neighbouring loops were combined in one Tx channel [see Fig. 4(a)]. For example, the antisymmetric L-shaped Tx channel (${T}_{x2}$) which have a common conductor and a SDC was formed by the combination of the two L-shaped elements 1 and 4. Likewise, the Tx channel (${T}_{x3}$) was formed by the combination of the two L-shaped elements 2 and 3. The same applies to the other antisymmetric L-shaped Tx channel (${T}_{x6}$ and ${T}_{x7}$), which was formed by the combination of the two elements 8 & 12 and the elements 7 & 11, respectively. The same applies to the other antisymmetric rectangular channels (${T}_{x5}$ and ${T}_{x8}$) were formed by the combination of the two elements 14 & 16 and the elements 13 & 15, respectively. The 2Tx channels (${T}_{x1}$ and ${T}_{x4}$) were formed by the combination of the two elements 6 & 10 and the elements 5 & 9, respectively. The array was connected to a Tx/Rx switch and 16-preamplifier interface (Rapid Biomedical GmbH, Rimpar, Germany) via four plugs (ODU GmbH & Co. KG, Mühldorf a. Inn, Germany) [see Fig. 4(h)]. Each plug was connected to four loop elements of the array via four 50 Ω coaxial cables K-02252-D-60 (Huber + Suhner, Herisau, Switzerland). The Tx/Rx interface was connected to the pTX system via two compatible Siemens Rx plugs and one Tx pTx plug (ODU GmbH & Co. KG, Mühldorf a. Inn, Germany). PTx ${B}_{1}^{+}$-shimming for varying both amplitudes/phases for the 8Tx/16Rx mono-surface array was performed using software provided by the vendor of the MRI system in the pTx mode. The 8Tx/16Rx mono-surface array was compared to an 8Tx/16Rx pTx transceiver human cardiac array prototype (Rapid Biomedical GmbH, Rimpar, Germany). The 8Tx/16Rx human cardiac array prototype consists of two sections, each comprising 8 loop elements with one central element, surrounded by seven elements, forming a circular shape coil arrangement^55^. Figure 4(f–h) shows the prototype of the dedicated 8Tx/16Rx mono-surface pig array loaded with the pig body phantom connected to the interface and the 7 T MRI system.

### Array characterization

#### S-matrix measurements

After obtaining the initial capacitor values from RF-circuit co-simulations using CST-DS, the final values of matching, tuning and decoupling capacitors were optimized on bench-top measurements using the cadaver of 46 kg pig as the load. The S-matrix was measured using an E5080A 4-port Vector Network Analyzer (VNA) (Keysight Technologies, Santa Rosa, CA, USA). For each resonant element, one solenoid cable trap was designed to remove the unbalanced surface current and cable resonance on the coaxial cables.

#### Relative HW phases

The implemented HW phases were obtained using an in-house developed MATLAB algorithm as described in^50^. To adjust the phase shift between the 16 loops, individual low pass π-network discrete phase shifter (PS) circuits were designed. The PS consists of two equal capacitors and one nonmagnetic and high quality factor inductor (Coilcraft, Inc., Silver Lake Road, Cary, IL). All PS circuits were inserted between cable traps and loops. The measured relative HW phases in the pig body phantom are shown in Table 2. To validate the optimal vectors PV1 and PV2, the phases are adjusted by coaxial cables and connected via BNC connectors to the interface as shown in Fig. 4(h). The coaxial RF cable phases were computed account of HW phases and with rounding the resulted phase to the nearest k·22.5°, where k is integer number [see Table 2].

### MRI measurements

All MR measurements were performed on a 7 T whole-body MAGNETOM Terra scanner (Siemens Healthineers, Erlangen, Germany) equipped with 8-channel RFPA (2 kW/channel) in pTx mode. All animals used in this work were German Landrace pigs obtained from Heinrichs Tierzucht GmbH, Heinsberg, Germany.

The two pig cadavers for coil optimization and testing were provided for MR-measurements immediately after usage in the approved project 55.2 DMS 2532–2–664 (Regierung Unterfranken, Germany). Euthanasia was performed with an intravenous application of 150-mg/kg pentobarbital under isoflurane anesthesia with fentanyl analgesia. The final and optimized version of the array was used for the *in-vivo* scans in agreement with the aims of the project named above. All experiments were performed in accordance with relevant guidelines and regulations.

#### Phantom MRI measurements

Phantom MR-experiments were performed using a 20 cm diameter spherical phantom and a pig body phantom (internal dimensions: maximum height in *y*-axis = 23 cm, maximum width in *x*-axis = 25 cm, length in *z*-axis = 30 cm, and with 1.0 cm thickness) to validate the ${B}_{1}^{+}$ field simulations [see Fig. 4(c–e)]. We selected the spherical phantom to test the worst-case scenario for loading conditions with side elements not loaded properly by the sphere. The pig body phantom was designed in-house and constructed from Plexiglas (Kunststoff Acryl Design GmbH, Essen, Germany). The composition of the phantom contents was: water (12.56 kg), PVP (5.96 kg), and salt NaCl (172 g), which results in volume of about 16 L and the electrical properties are ${{\rm{\varepsilon }}}_{{\rm{r}}}$ ≅ 58 and ${\rm{\sigma }}$ ≅ 0.77 S/m. The measurements of the ${B}_{1}^{+}$ spatial distribution in both phantoms and pig cadaver experiments were performed using the saturated double flip angle method (SDAM) based on a GRE sequence. The GRE-SDAM measurement parameters were: TE/TR = 1.8/4000 ms, pixel resolution 2 × 2 mm, slice thickness = 4 mm for the transversal and for coronal views. The images for FA-map reconstruction were acquired in two successive measurements with doubling voltage of the excitation RF-pulse. The actual FA maps were reconstructed using an in-house developed MATLAB script.

#### MRI measurements with pig cadavers

Performing measurements in a fresh pig cadaver allows consistent conditions for high-resolution scans using varying acceleration factors, while avoiding cardiac and respiratory motion. In particularly, the FA-maps can be acquired using ground-truth SDAM methods which are not feasible *in-vivo*, since the long repetition times needed to exclude effects of ${T}_{1}\,\,$weighting, result in long acquisition times. The total measurement time was about two hours covering the preparatory pulse sequences, anatomical GRE sequence, FA maps, SNR maps, noise correlation, and the g-factor maps. The pig cadavers were placed head first in dorsal recumbence inside the scanner. The position of the heart was estimated to fit approximately in the center of the coil. The sequence parameters for characterization of SNR and parallel imaging capability of the array in cadaver experiments were based on the standard GRE CINE protocol. The sequence parameters were: TR/TE = 69.52/4.07, FA = 45°, pixel size = 0.3 mm × 0.3 mm, number of averages = 8, FOV = 320 × 320 mm and a slice thickness of 6 mm. The analysis of SNR was carried out on SA and LA images. Varying parallel acceleration factors from R = 2–6 resulted in TEs/TRs of 4.7/69.52 ms, 4.7/78.21 ms, and 4.7/78.75 ms. Parallel imaging methodology used for accelerating MR image acquisitions was Generalized Auto-Calibrating Partially Parallel Acquisition (GRAPPA). The SNR in the images was calculated as the signal intensity in a given voxel divided by the image noise. The noise was determined as the standard deviation within a ROI placed in air. A correction factor was applied according to^56^. In addition, the SNR in myocardial tissue in dependence of the acceleration factors was assessed within four ROIs placed in the left ventricle for both SA and LA views. The GRE-SDAM measurement parameters for both cadaver pig measurements were: TE/TR = 1.8/4000 ms, pixel resolution 2 × 2 mm, slice thickness 6 mm, and number of slices = 6. The g-factor characterizations for both cadaver pig measurements were done using vendor-provide protocol and reconstruction pipeline (“coilutil”). The measurements sequence was GRE with TR = 3.9/TE = 10 ms FA = 15°, number of averages = 10, slice thickness = 6 mm, spatial resolution in-plane 0.5 × 0.5 mm. For comparison the FA-maps and g-factor measurements were done with the 68 kg cadaver using 8TX/16RX human cardiac array prototype.

#### *In-vivo* MR measurements

The pig for an *in-vivo* measurement was anesthetized using isoflurane with additional fentanyl analgesia and artificially ventilated using an MR-compatible ventilator (Draeger, Germany). Ventilation parameters, heart rate, sPO2 and rectal temperature were monitored through whole session. The heart rhythm monitoring was performed using ECG (vendor integrated) and acoustic triggering system (ACT, MRI.Tools, Berlin, Germany) connected to the external triggering input. Outside of the scanner bore the ECG and ACT system show very close heart rate and duration of RR-interval (as measured by scanner system). Inside the bore the ECG signal was significantly distorted by magneto-hydrodynamic-effect and, thus, ACT system was used for the triggering of CINE acquisition. The measurement parameters of the GRE CINE pulse sequence were: TR/TE = 44/2.9 ms, FA = 35°, pixel resolution in-plane 0.6 mm × 0.6 mm, slice thickness 6 mm; 30 heart beating phases was retrospectively reconstructed using 21, 14, 12, and 9 RR-cycles acquired with parallel imaging GRAPPA acceleration factors R = 2, 3, 4, and 6, respectively. The *in-vivo* measurement was performed using the optimal phase vector PV2.

## Supplementary information

Supplementary Data.
