# Supplementary material for: A Novel Mono-surface Antisymmetric 8Tx/16Rx Coil Array for Parallel Transmit Cardiac MRI in Pigs at 7T
Source: Sci Rep. 2020 Feb 20;10:3117. doi: 10.1038/s41598-020-59949-6 (PMC7033245; doi:10.1038/s41598-020-59949-6)
Supplement: Supplementary file 1 — Supplementary Data. [file 41598_2020_59949_MOESM1_ESM.pdf]

A Novel Mono-surface Antisymmetric 8Tx/16Rx Coil Array for Parallel Transmit Cardiac MRI in Pigs at 7T

Ibrahim A. Elabyad, Maxim Terekhov, David Lohr, Maria R. Stefanescu, Steffen Baltes, and Laura M. Schreiber

Chair of Cellular and Molecular Imaging, Comprehensive Heart Failure Center (CHFC), University Hospital Wuerzburg, D-97078 Wuerzburg, Germany

Table S1. Simulated (yellow) and measured S-Matrix in dB for the mono-surface array loaded with a 20 cm diameter spherical phantom with ε_r = 59.3 and σ = 0.79 S/m.

El.#	1	2	3	4	5	6	7	8	9	10	11	12	13	14	15	16	El.#
	−12	−18	−16	−12	−21	−13	−25	−26	−22	−18	−27	−23	−24	−25	−26	−38	1
		−16	−16	−18	−16	−26	−17	−22	−16	−22	−24	−32	−21	−25	−33	−23	2
1	−10		−17	−18	−16	−26	−10	−26	−16	−22	−15	−28	−21	−25	−33	−27	3
2	−17	−10		−16	−24	−16	−37	−13	−22	−10	−31	−12	−29	−25	−34	−34	4
3	−18	−12	−14		−23	−20	−13	−27	−11	−27	−48	−26	−15	−42	−17	−40	5
4	−12	−18	−21	−14		−26	−26	−25	−28	−15	−42	−24	−27	−19	−30	−19	6
5	−20	−13	−16	−27	−21		−9.0	−35	−18	−30	−10	−30	−14	−27	−14	−28	7
6	−13	−20	−27	−16	−20	−21		−12	−32	−9.0	−47	−10	−30	−13	−29	−14	8
7	−24	−32	−12	−28	−16	−28	−21		−10	−30	−23	−29	−10	−31	−9.0	−35	9
8	−32	−24	−28	−12	−28	−16	−30	−22		−10	−40	−14	−43	−12	−37	−11	10
9	−19	−17	−9.0	−23	−15	−23	−11	−25	−15		−9.0	−31	−9.0	−31	−14	−28	11
10	−17	−19	−23	−9.0	−23	−15	−25	−11	−27	−15		−11	−34	−9.0	−40	−12	12
11	−22	−23	−15	−28	−19	−25	−8.0	−29	−16	−40	−17		−8.0	−33	−8.0	−41	13
12	−23	−22	−28	−15	−25	−19	−29	−8.0	−40	−16	−34	−17		−14	−44	−9.0	14
13	−22	−21	−24	−29	−25	−32	−12	−25	−10	−29	−8.0	−40	−15		−10	−31	15
14	−21	−22	−29	−25	−32	−25	−25	−12	−29	−10	−40	−8.0	−30	−15		−15	16
15	−27	−33	−19	−28	−18	−42	−10	−29	−8.0	−41	−20	−29	−8.0	−43	−22		
16	−33	−27	−28	−19	−43	−18	−29	−11	−41	−9.0	−29	−20	−43	−8.0	−39	−22	
	1	2	3	4	5	6	7	8	9	10	11	12	13	14	15	16	

Table S2. Measured S-Matrix in dB for the mono-surface array loaded with a 46 kg cadaver pig.

El.#	1	2	3	4	5	6	7	8	9	10	11	12	13	14	15	16
1	−17															
2	−13	−23														
3	−18	−12	−19													
4	−15	−20	−18	−15												
5	−19	−11	−13	−21	−28											
6	−11	−26	−24	−15	−19	−24										
7	−28	−15	−11	−37	−16	−33	−20									
8	−23	−25	−24	−13	−27	−21	−57	−18								
9	−22	−19	−23	−25	−12	−35	−21	−33	−15							
10	−20	−20	−18	−15	−35	−15	−30	−16	−28	−20						
11	−39	−26	−18	−52	−22	−30	−13	−40	−32	−33	−17					
12	−34	−37	−48	−20	−30	−40	−42	−13	−39	−20	−38	−27				
13	−31	−25	−36	−32	−21	−32	−16	−40	−15	−38	−12	−44	−25			
14	−26	−31	−26	−27	−35	−24	−40	−17	−37	−16	−36	−12	−44	−21		
15	−34	−40	−34	−47	−35	−38	−22	−45	−12	−43	−22	−52	−16	−48	−22	
16	−37	−30	−34	−37	−37	−22	−40	−19	−44	−13	−39	−17	−44	−12	−45	−22

Table S3. Computed optimal amplitudes/phases within the pig body phantom for the 8Tx channels after on-scanner pTx B_1^+ using the vendor integrated pTx shimming algorithm.

pTx Channel #	Magnitude	Phase [°]
T_{x1}	0.40	00.00
T_{x2}	0.41	33.68
T_{x3}	0.42	−98.95
T_{x4}	0.41	−41.85
T_{x5}	0.41	−12.14
T_{x6}	0.41	−69.62
T_{x7}	0.40	−33.25
T_{x8}	0.41	−19.95
